# *In‐Operando* FTIR Study to Investigate the Effect of Varying Lithium Salts on Solid Electrolyte Interface (SEI) Evolution in Lithium Metal Batteries

**DOI:** 10.1002/advs.202523503

**Published:** 2026-03-16

**Authors:** Samia Rahman, Rhyz Pereira, Jantakan Nedsaengtip, Vibha Kalra

**Affiliations:** ^1^ RF Smith School of Chemical and Biomolecular Engineering Cornell University Ithaca New York USA; ^2^ Department of Chemical and Biological Engineering Drexel University Philadelphia Pennsylvania USA

**Keywords:** FTIR, operando, salt, solid electrolyte interface (SEI), spectroscopy

## Abstract

Lithium Metal Batteries (LMBs) offer exceptional energy density but suffers from unstable Solid Electrolyte Interface (SEI). This study employs *in‐operando* Fourier transform infrared (FTIR) spectroscopy, coupled with *post‐mortem* XPS, to deconstruct the real‐time evolution of the SEI in Li||Li symmetric cells at a practical current density of 3 mA/cm^2^. We investigate three lithium salts in a DME: DOL solvent – 1m LiTFSI, 1m LiClO_4_, and 1m LiDFOB. *In‐ operando* FTIR reveals distinct, salt‐dependent SEI formation dynamics. The results reveal that LiTFSI interacts instantaneously with the lithium surface, creating a robust, hybrid SEI composed of both organic (e.g., ROLi, ROCO_2_Li) and inorganic species (e.g., Li_2_O, Li_3_N, LiF). This stable interface correlates with superior electrochemical performance, exhibiting the lowest and most stable overpotential. In contrast, LiClO_4_ shows a delayed interaction, with SEI formation commencing minutes into plating and featuring decomposition products such as LiCl. The LiDFOB electrolyte proves to be the least effective, yielding an unstable, organic‐rich SEI that results in high overpotential and poor performance. This study establishes a clear correlation between SEI composition and electrochemical performance, offering a molecular‐level understanding to inform rational electrolyte design and salt selection strategies for stable lithium metal batteries.

## Graphical abstract

A clear correlation between electrolyte salt chemistry and Solid Electrolyte Interface (SEI) performance in lithium metal batteries is established using in operando FTIR spectroscopy. This study shows that LiTFSI instantaneously forms a robust, inorganic organic hybrid SEI (LiF, Li_3_N, Li_2_O, ROCO_2_Li, ROLi) that minimizes overpotential, while less effective salts yield unstable, organic‐rich interphases and poor cycling performance.

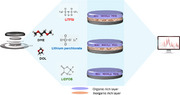

## 1 Introduction

The growing demand for high‐energy‐density batteries has driven interest in technologies beyond lithium‐ion batteries (LIBs), which are now approaching their theoretical performance limits [1]. Lithium metal, with its ultrahigh theoretical capacity (3860 mAh g^−^^1^) and lowest electrochemical potential (−3.04 V vs SHE), is known to be the holy grail of anode materials in next‐generation batteries [2, 3]. When paired with high‐capacity cathodes such as sulfur, oxygen, or transition metal oxides, lithium metal batteries (LMBs) offer exceptional energy density [4, 5, 6, 7]. However, challenges such as dendrite growth and dead inactive lithium formation lead to poor cycling stability and safety concerns. A key strategy to address these issues is optimizing electrolyte formulations to promote the formation of a stable and uniform solid electrolyte interface (SEI).

Electrolyte components, both the solvent and salt, play a critical role in determining the capacity, efficiency, and long‐term stability of lithium metal batteries [7]. In particular, the choice of lithium salt significantly influences the interfacial chemistry at the lithium surface, fundamentally altering the SEI's composition, formation kinetics, and mechanical stability [8]. While numerous studies have explored salt selection through cycling performance and postmortem analysis, the specific mechanisms by which different salts affect SEI properties and impact long‐term stability remain under debate [8, 9, 10, 11, 12]. In this regard, operando techniques can help capture the real‐time interfacial reactions and provide deeper insights into these complex electrolyte–electrode interactions.

A comprehensive understanding of the composition and structure of SEI is limited due to its complexity and dynamic behavior during cycling. To investigate these phenomena, a suite of different in situ and *in‐operando* techniques have been leveraged. These techniques allow characterization under static (in situ) and dynamic operational conditions (*in‐operando)*. Since SEI formation is a surface‐driven phenomenon, surface‐sensitive techniques such as XPS and FTIR‐ATR are extensively used to probe its chemical composition [13]. Complementary insights into its electrochemical and structural properties are obtained through other techniques; *operando* electrochemical Impedance Spectroscopy (EIS) provides critical data on interfacial resistance, while in situ and operando X‐ray diffraction (XRD) monitors crystalline and structural changes. Collectively, these techniques provide a clear view of the SEI evolution and dynamics. However, the utility of many of these studies is often limited by simplified electrochemistry, such as single salt electrolyte, low current density, or slow scan rate, leaving questions such as the influence of varied electrolyte formulations on SEI unanswered.

In situ and *in‐operando* FTIR spectroscopy can offer valuable insights into the evolution of chemical bonds and molecular structure at electrochemical interfaces. FTIR is particularly sensitive to organic and inorganic polar functional groups such as carbonates, ethers, alkoxides, and fluorinated species, making it a powerful tool to track solvent decomposition, salt degradation, and interphase formation in real time. It complements other surface‐sensitive techniques like XPS and ToF‐SIMS by enabling time‐resolved, non‐destructive monitoring of chemical processes under actual operating conditions. *In operando* FTIR spectroscopy has been used in lithium‐ion batteries to explore the electrochemical interaction and dynamic behavior on the electrode‐ electrolyte interface [14, 15, 16]. To our knowledge, only a handful of studies have developed and investigated *operando* FTIR to study lithium metal batteries. Examples include studies by Olana et al., in 2022 and Wu et al., in 2023 [17, 18]. Both studies focused on the effect of LiNO_3_ as an additive and its impact on LiTFSI decomposition on the lithium surface. Olana et al., conducted in situ DRIFTS study at different potentials, capturing spectral snapshots under a slow scan rate of 0.1mV/s. Wu et al., studied the effect of LiNO_3_ on dynamic SEI formation cycled at 0.5mV/s. Their findings suggest LiNO_3_ contributes to a more stable SEI. While these studies are valuable and represent important early steps in conducting real‐time FTIR investigations in lithium metal batteries, they remain limited by slow scan rate CV/LSVs and a narrow focus on a single electrolyte additive—highlighting the need for broader and more systematic studies. Our work represents direct progression from these important findings, broadening the chemical scope to include a more diverse range of electrolyte systems and investigating SEI dynamics under the more demanding, high‐rate cycling protocols relevant to practical applications. In this work, we investigate the SEI formation mechanism and dynamics for three Li salts: lithium bis(trifluromethane) sulfonimide (LiTFSI), lithium perchlorate (LiClO_4_), and lithium difluoro (oxalato) borate (LiDFOB). The operando cells were cycled at a high current density of 3mA/cm^2^ that resembles the practical operating condition of LMBs [19, 20]. Our work provides molecular‐level insights into how lithium salt fundamentally shapes SEI dynamics and battery stability in the lithium metal system.

Ether‐based electrolytes, particularly the solvent system of 1,2‐dimethoxyethane (DME) and 1,3‐dioxolane (DOL), are known for a more stable SEI compared to carbonate‐based ones [11]. LiTFSI is one of the most used salts in lithium metal batteries and is the primary salt for high‐energy‐density lithium‐sulfur (Li─S) batteries owing to its high ionic conductivity, thermal stability, and compatibility with ether‐based electrolytes [21]. However, it suffers from serious issues, particularly the corrosion of the aluminum current collector at high voltage [22]. LiClO_4,_ on the other hand, has been extensively studied for lithium batteries because of its higher electrochemical stability, low moisture sensitivity, and suitable current collector passivation [23]. LiDFOB is a popular secondary salt/ additive used mainly with carbonate electrolyte and has been recognized for stabilizing the SEI in lithium‐ion batteries [24, 25, 26]. Additionally, previous literature has reported poor performance of LiDFOB salt with ether‐based electrolyte with no real‐time mechanistic explanation [9]. Here in this study, we track the dynamic changes using advanced *operando* FTIR‐ATR spectroscopy. We systematically investigated three widely used salts, LiTFSI, LiClO_4,_ and LiDFOB under a high, commercially relevant current density of 3mA/cm^2^. Our real‐time spectroscopic results show that LiTFSI forms a stable, mixed organic inorganic SEI immediately upon contact with lithium, enabling low and stable overpotentials. In contrast, LiClO_4_ displays delayed reactivity and forms a LiCl‐rich SEI, while LiDFOB leads to an unstable, organic‐dominated SEI associated with poor electrochemical performance. These findings highlight the critical role of lithium salt selection in shaping interfacial chemistry and guiding the design of high‐performance electrolytes for lithium metal batteries.

## 2 Experimental Methods

### 2.1 Electrolyte Preparation

1:1 volumetric mixture of DME (Sigma Aldrich, anhydrous, 99.5%, inhibitor‐free), DOL (ACROS Organics, 99.8%, anhydrous) was made inside an Argon‐filled glovebox with water and oxygen level < 0.1ppm. We will refer to this 1:1 volumetric mix of DME and DOL solvent as DMEDOL throughout the study. Electrolyte with 1M LiTFSI (Sigma Aldrich, 99.99% trace metal basis), LiClO_4_ (Thermo Scientific Chemicals, 99%, battery grade), LiDFOB (MSE supplies, high purity 99.9%) salt in DME, DOL (1:1) was prepared by adding appropriate amounts of salt and solvent in an Ar‐filled glovebox. We will refer to these electrolytes as DMEDOL – TFSI, DMEDOL – PC and DMEDOL–DFOB electrolytes.

### 2.2 Cell Fabrication and Electrochemical Testing

All electrochemical testing was done in 2032 coin cells. Galvanostatic charge discharge (plating and stripping) on the Li|| Li symmetric cells were conducted at 3 mA/cm^2^ current density. Cells were fabricated using two 15.6mm diameter, 0.25 mm thickness lithium chips as both working and reference electrodes, separated with a Celgard 2325 separator. 25 µL of electrolyte was used in each cell. A Neware battery testing system was used for coin cell testing and analysis. To characterize the interfacial kinetics during cycling, electrochemical impedance spectroscopy (EIS) was performed on Li||Li symmetric cells using a Biologic potentiostat. Measurements were recorded at open circuit voltage (OCV) immediately after assembly, at specific intervals (15, 30, 60, and 120 min) during the lithium plating process, and finally after lithium stripping. The impedance spectra were acquired over a broad frequency range from 1MHz to 1mHz to capture both high‐frequency interfacial processes and low‐frequency diffusion dynamics. The lithium working electrode was analyzed using X‐ray photoelectron spectroscopy (XPS) after the initial plating and again after five complete plating/stripping cycles. XPS and scanning electron microscopy (SEM) analyses were performed at the Cornell Center for Materials Research (CCMR). A Thermo Fisher Scientific Nexsa G2 was used for XPS, and a ZEISS Gemini 500 was used for SEM.

### 2.3 In‐Operando FTIR Cell Assembly

**SCHEME 1:**
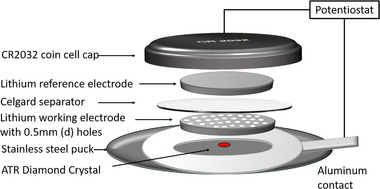
Schematic of *in‐operando* FTIR coin cell setup on the ATR diamond.

The *in‐operando* FTIR‐ATR cell was built directly on the ATR accessory. Scheme 1 shows the schematic of the *in‐operando* FTIR coin cell setup featuring the ATR diamond crystal. We used a Thermo‐fisher Scientific Nicolet iS50 FTIR spectrometer, equipped with an extended range ATR accessory and with a deuterated triglycine sulfate (DTGS) detector. To assemble the operando FTIR cell, the ATR puck was transferred into an argon‐filled glovebox, where a lithium||lithium symmetric coin cell was built directly on the diamond crystal. The cell used 25 µL of electrolyte, two 10 mm lithium disc electrodes, and a Celgard 2325 separator. The working electrode had small (0.5 mm) holes to enhance SEI detection. Instead of a stainless‐steel spacer, a layered Ni foam was used to maintain proper contact on the cell. The assembly was sealed with a 2032‐coin cell top, vacuum grease, and Kapton tape before being taken outside the glovebox. It was then connected to a Gamry Reference 3000 Potentiostat, with the stainless‐steel puck as the current collector and an Al foil strip for external connection. Finally, the FTIR instrument's pressure anvil was applied to ensure proper contact during measurement.

Lithium plating and stripping in the Li||Li cell was conducted at a current density of 3 mA/cm^2^. A baseline spectrum was first collected at open circuit voltage (OCV) before applying current, followed by continuous spectral collection throughout the plating and stripping process. To minimize the complexity of the *operando* FTIR data, spectra collected at OCV, 15, 30, 60, and 120 min have been chosen as the representative spectra instead of all the spectra collected during cycling. Spectral data were processed using OMNIC software, with advanced ATR correction and baseline corrections applied. The extended‐range FTIR‐ATR recorded spectra across 400–4000 cm^−^^1^, but since most characteristic peaks appear within 400–2000 cm^−^^1^, this range was primarily used for analysis.

To establish baseline measurements, FTIR spectra of pure DME, pure DOL, and their 1:1 mixture were first collected. A custom operando FTIR cell was assembled inside an argon glovebox to prevent air exposure during sample handling and spectral acquisition. Baseline spectra of the three electrolytes—each containing LiTFSI, LiClO_4_, or LiDFOB salt in DMEDOL, referred to as DMEDOL – TFSI, DMEDOL – ClO_4_, DMEDOL – DFOB, respectively, were also recorded. To isolate the effect of the solvent alone, Li metal chips were immersed in DMEDOL without applied current in a sealed environment, and FTIR spectra were collected immediately and after 1 hour to observe any spontaneous interfacial interaction between lithium and the solvent mixture.

## 3 Results and Discussion

### 3.1 FTIR – ATR Spectra of Solvent and Electrolytes as Reference

**FIGURE 1:**
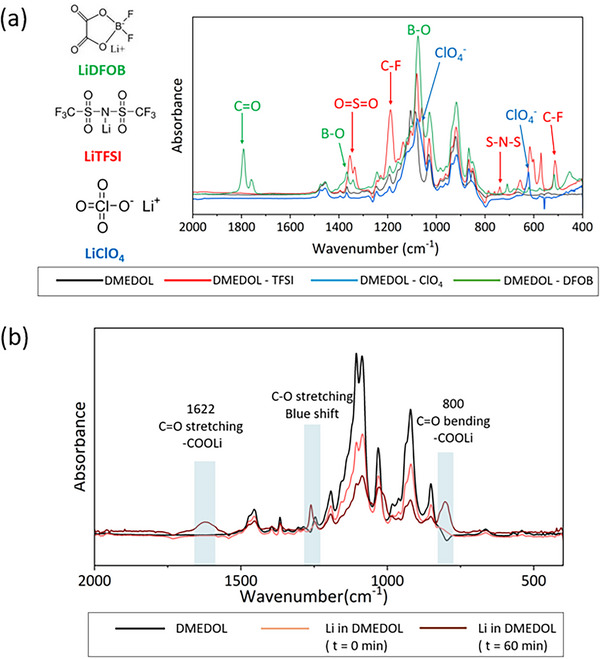
(a) spectra of DMEDOL solvent, DMEDOL – TFSI, DMEDOL – DFOB, DMEDOL – ClO_4_ electrolyte (b) FTIR spectra of Li metal chips in DMEDOL solvent (no salt).

To establish a clear baseline for the operando experiments, the initial spectra of three electrolytes were studied prior to electrochemical cycling. Each electrolyte containing either LiTFSI, LiClO_4,_ or LiDFOB salt in DMEDOL solvent was found to show a unique spectral fingerprint, shown in Figure 1a. For comparison, we provide the spectrum of the DMEDOL solvent without salt. The characteristic signature of LiTFSI comes from O═S═O, C─F, and S─N─S bonds [21], LiClO_4_ from Cl─O bond vibration, which is the characteristic peaks for perchlorate anion [27], and C═O, B─O, and B─F are the major peaks from LiDFOB salt [25]. The spectra illustrate the chemical diversity of the salts. Table 1 shows a comprehensive summary of all peak assignments. For clarity, we have labeled the spectra with only the peaks arising from the salt.

**TABLE 1:** Characteristics of peaks from three electrolytes.

DMEDOL + LiTFSI		DMEDOL + LiClO4		DMEDOL + LiDFOB	
1354	O═S═O stretching[28]	1103	Cl─O stretching[29]	1791	C═O stretching[30]
1333	O═S═O stretching[28]	622	Cl─O stretching[29]	1759	C═O stretching[30]
1228	C─F stretching[18]			1367	B─O stretching[31]
1185	C─F stretching[18]			1244	B─F stretching[32]
1080	C─O stretching[33]			1075	B─O, C─O stretching[34]
741	S─N stretching[28]			708	B─O stretching[35]
600, 616	S─N─S stretching[36]				

Alongside the major peaks assigned to the salts, prominent peaks from the solvent are also visible. A detailed peak assignment for the pure DME and DOL solvents, as well as their 1:1 mixture DMEDOL, is provided in Figure S1.

To isolate the interaction between lithium metal and the solvent, we performed a salt‐free control experiment. Specifically, we immersed lithium metal in the DMEDOL solvent (no salt) in a sealed cell and collected spectra immediately and again after 1 hr. Figure 1b shows these spectra compared to that of the pure solvent.

No spectral changes were observed upon initial contact of DMEDOL solvent with the Li metal disc. However, after 1 hr, two new peaks emerge for C═O stretching at 1622 cm^−1^ and C═O bending at 800 cm^−1^ suggesting the formation of lithiated ─COO compounds such as HCOOLi, CH_3_COOLi, and CH_3_CH_2_COOLi [33]. The blue shift of C─O bond from 1245 to 1260 cm^−1^ suggests an increase in the bond strength. This observation is counter‐intuitive as one would expect weakening of the C─O bond (or red shift) due to Li^+^ ion coordination. DFT calculations by Liu et al., showed that although the C─O bond initially weakens with Li+ solvation, increasing Li+ concentration in the solvation complex reduces the O─Li solvation radius, slightly strengthening the C─O bond and shifting it to higher frequency [37].

### 3.2 *In‐Operando* FTIR Characterization During Cell Cycling

#### 3.2.1 DMEDOL – TFSI Electrolyte

**FIGURE 2:**
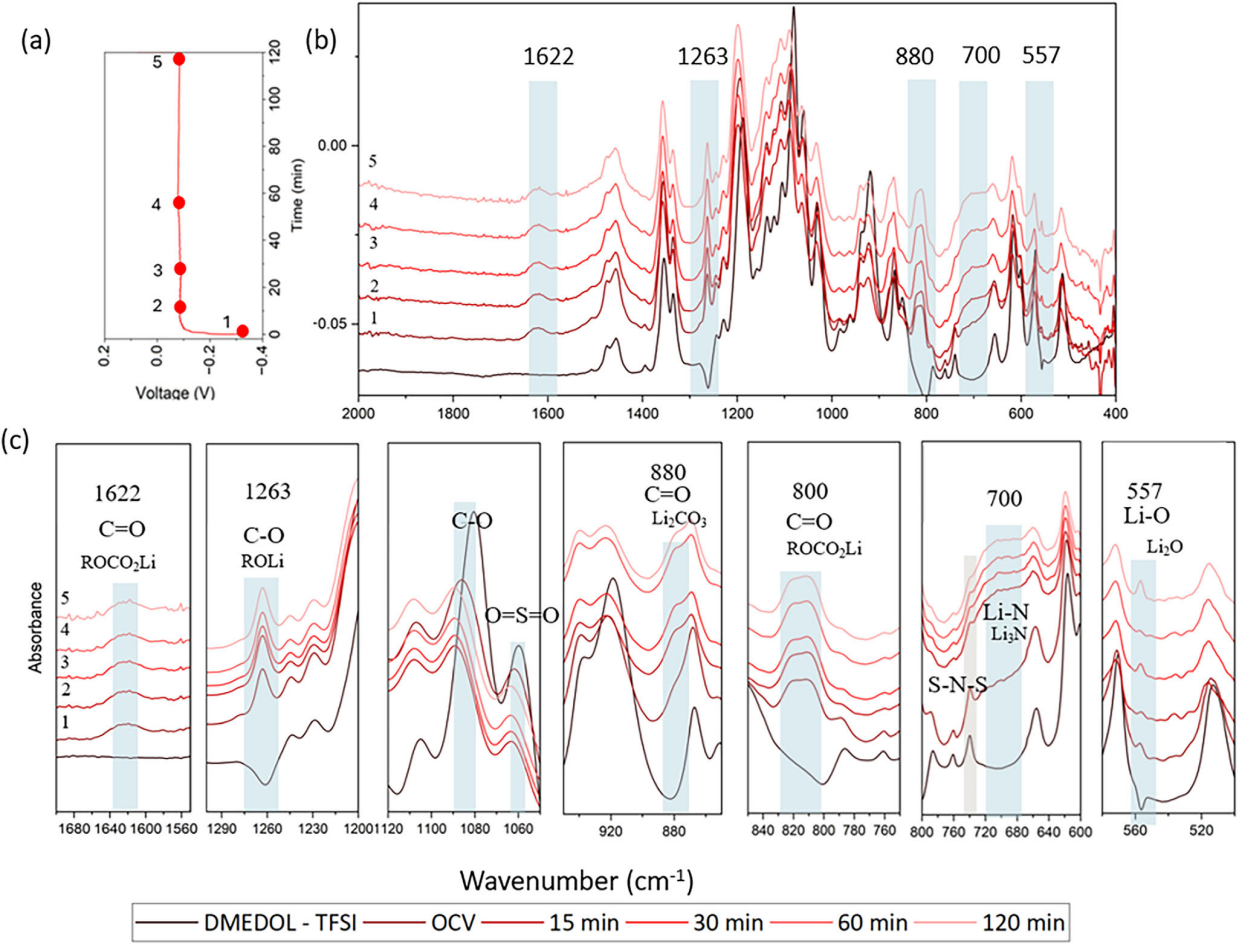
*In‐operando* FTIR spectra during first plating with DMEDOL‐TFSI electrolyte (a) Voltage vs time (min) plot during plating (b) FTIR spectra of DMEDOL – TFSI, OCV, 15, 30, 60, and 120 min into the plating (c) Magnified views of key vibrational bands, highlighting the emergence and evolution of SEI‐related species.

To investigate the dynamic evolution of SEI, *in‐operando* FTIR cells were assembled in a Li‖Li symmetric configuration using DMEDOL solvent containing one of three lithium salts: 1 M LiTFSI (DMEDOL – TFSI), 1 M LiClO_4_ (DMEDOL – ClO_4_), and 1 m LiDFOB (DMEDOL – DFOB). Each cell underwent a 2‐h plating/stripping cycle. Figure 2a shows the voltage vs. current data of the operando electrochemical cell, and Figure 2b shows the FTIR spectra collected at different time points. Finally, Figure 2c zooms into the specific spectral changes that we observe during plating on the working electrode. As shown in Figure 2b, compared to the DMEDOL – TFSI electrolyte spectrum, significant spectral changes occur at open‐circuit voltage (OCV), prior to current application, indicating rapid and spontaneous SEI formation upon initial contact with the lithium surface.

Among the emerged peaks at OCV, the 1622 and 800 cm^−1^ are attributed to C═O stretching and C═O bending, respectively, from lithiated ‐COO compounds, consistent with our salt‐free control experiment. Additionally, the presence of LiTFSI facilitates a more complex solvent decomposition leading to additional new peaks at 1263, 880, and 557 cm^−1^ that can be attributed to C─O stretching from lithiated oligomers ─C─O─Li (ROLi species) [38], C═O in plane bending from CO_3_^2−^ group in Li_2_CO_3_ [39, 40, 41] and asymmetric stretching of Li─O from Li_2_O [33].

Upon applying current, the cell was subjected to a 2 h plating, maintaining a stable voltage profile shown in Figure 2a. During the dynamic plating process, decomposition of TFSI^−^ anion was observed. The S─N─S stretching vibrational peak at 741 cm^−1^ significantly reduces in intensity over time, suggesting breakage of this bond. In parallel, we see the emergence of a small and wide Li─N stretching peak at 700 cm^−1^ suggesting the formation of Li_3_N [33]. This observation provides direct spectroscopic evidence for the transformation of salt anion into inorganic SEI components during cycling.

This reaction also causes blue shifts in the O═S═O and C─F stretching modes. The O═S═O bond shifted from 1057 cm^−1^ to 1062 cm^−1^, likely due to Li^+^ coordinating with the oxygen atoms without breaking the O═S═O bond [17]. This coordination withdraws electron density, making the sulfur more electropositive and increasing the double‐bond character of the S═O bonds, leading to a higher vibrational frequency. A similar shift is observed for the C─F bond, which moves from 570 to 573 cm^−^^1^. Both shifts may also be related to the partial cleavage or rearrangement of the S─N─S backbone in the TFSI anion. From the spectral changes, it is seen that the major SEI components formed from LiTFSI reduction include lithium alkoxides (ROLi), lithium alkyl carbonates (ROCO_2_Li), lithium oxide (Li_2_O), lithium nitride (Li_3_N), and lithium carbonate (Li_2_CO_3_).

Li anode is expected to have a thin layer of electrolyte between the Li metal and FTIR – ATR diamond crystal. And some of the SEI compound peaks could possibly be overlapping with the electrolyte spectra. To isolate SEI‐specific features, the spectrum of the DMEDOL – TFSI electrolyte was subtracted from all i*n‐operando* FTIR spectra. Figure S2 shows the electrolyte subtracted spectra at OCV and 15, 30, 60, and 120 min into the plating. Notably, like the non‐subtracted spectra, most peak changes in the subtracted spectra appear at open‐circuit voltage, with only the C─H out‐of‐plane bending mode at 920 cm^−^^1^ showing a gradual increase after 15 min of plating. This, along with the C─O stretching peak at 1032 cm^−^^1^, suggests the formation of polymerized DOL (poly‐DOL) within the SEI [42]. Li et al., proposed that in situ formation of poly‐DOL contributes to a flexible SEI capable of accommodating volume changes based on post‐mortem analysis and cell performance. Our work directly captures the emergence of polymeric ether‐related vibrations in‐operando. Meanwhile, inorganic components such as LiF, Li_2_O, and Li_3_N contribute to the SEI's chemical stability, ionic conductivity, and barrier properties. The synergistic effect of these two classes, mechanically resilient organics and ionically conductive, stable inorganics, is expected to promote a more robust and durable SEI, less prone to cracking or continuous restructuring during cycling [43].

**FIGURE 3:**
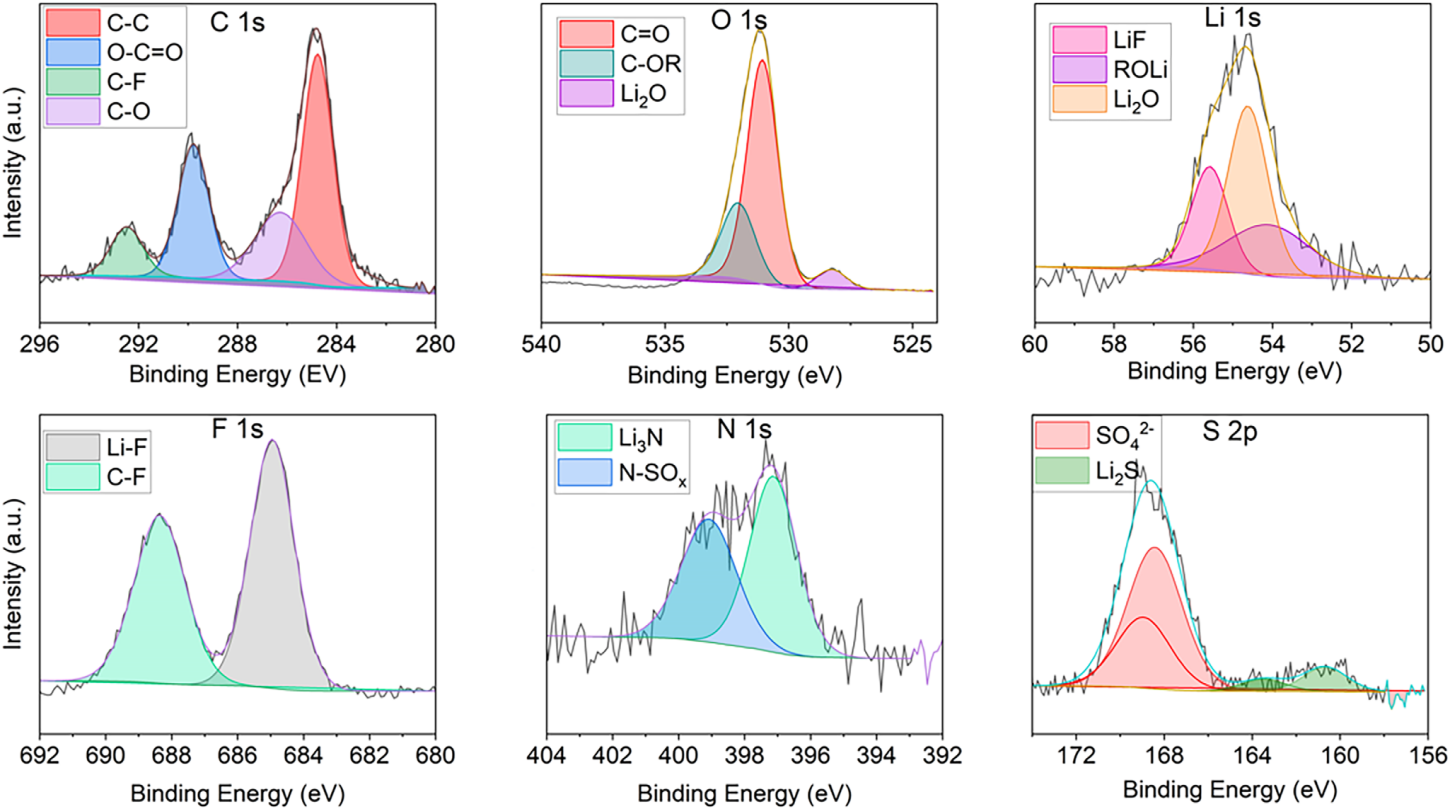
High‐resolution XPS spectra of various elements of lithium cycled in DMEDOL‐TFSI electrolyte.

To complement *in‐operando* FTIR data, we conducted XPS analysis on the cycled Li surface after 5 cycles, after plating. Figure 3 shows the C 1s, O 1s, Li 1s, F 1s, N 1s, and S 2p high‐resolution XPS images from Li metal surface. C 1s shows the presence of C─C (284.8 eV), O═C─O (289.7 eV), C─F (292.6 eV), C─O (286.4 eV), and C─F (292.6 eV) [44]. These components indicate the formation of polymeric carbon species, alkoxide (ROLi), carbonate species (Li2CO3 and ROCO2Li), and fluorinated carbon species from salt decomposition. XPS also confirms the presence of Li_2_O (528.3 eV in Li 1s spectra) [45], Li_3_N (397.1 eV in N 1s spectra) [46], Li_2_S (161.5 eV in S 2P spectra) [47] and Li_2_CO_3_ (531.2 eV in O 1s spectra) [48] and additionally shows the presence of Lithium fluoride, (LiF) at 68.5eV in F 1s spectra [49]. The detection of Li_2_S in the S 2p spectrum sgests the presence of sulfur‐containing species associated with partial decomposition of the TFSI^−^ anion at the lithium metal interface. Li_2_S is an electronically insulating inorganic component that may contribute to surface passivation when present alongside other SEI species. In the DMEDOL–TFSI system, the presence of Li_2_S together with LiF, Li_2_O, and Li_3_N points to a chemically heterogeneous inorganic SEI. The inability to observe LiF and Li_2_S with FTIR is because vibrational modes of LiF occur at low frequency below the spectrometer's detection range (<400), while the high crystal symmetry of Li_2_S makes it infrared inactive [50].

**SCHEME 2:**
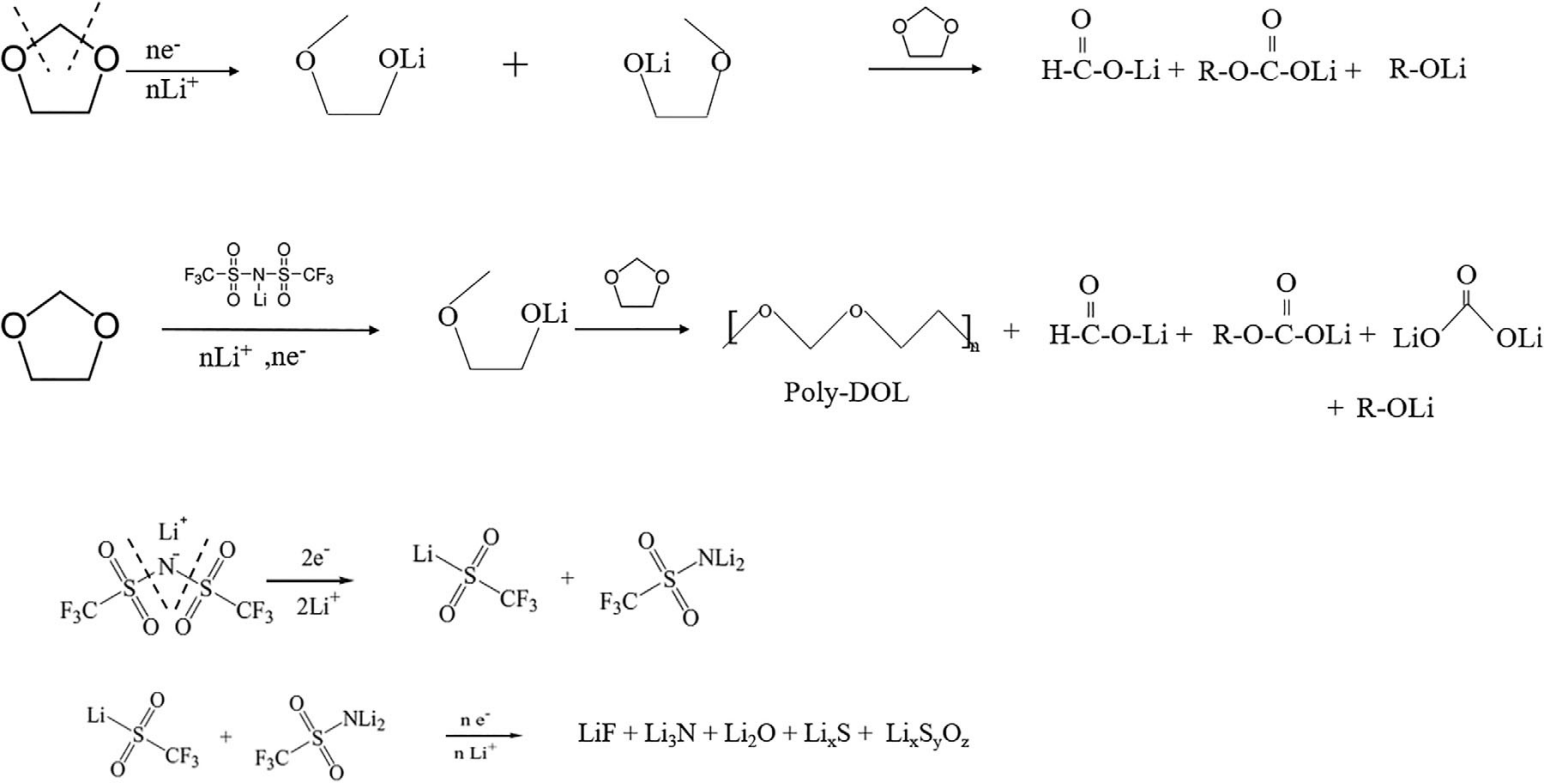
Proposed reaction mechanism of DMEDOL – TFSI electrolyte in lithium metal surface.

Based on our findings from in‐*operando* FTIR supported by *postmortem* XPS, the reaction mechanism that we propose for the interaction of LiTFSI salts and DMEDOL solvent with Li metal anode are shown in reactions (iii) and (iv) in Scheme 2.

In the absence of salt, DOL undergoes a nucleophilic ring‐opening reaction initiated directly by the lithium metal surface. The highly reducing nature of lithium provides electrons to electrophilic carbon in the DOL ring, breaking it open, resulting in the formation of lithium alkyl carbonates (ROCO_2_Li) and lithium alkoxides (ROLi) as SEI components. This reaction occurs spontaneously due to the strong driving force from lithium metal and generates organic SEI species. However, in the presence of salt, the increased concentration of Li^+^ in the system and the presence of anions that act as a radical initiator for the polymerization reaction promote polymerization. As a result, DOL undergoes chain growth, forming poly DOL in the SEI as directly observed via our operando FTIR spectra.

For the DMEDOL – TFSI electrolyte, *operando* FTIR data additionally shows the S─N─S bond breakage during reduction on the lithium metal surface. The schematic shows that under electron‐rich conditions, TFSI decomposes into reactive intermediates. The intermediates are chemically unstable, and their further breakdown leads to the formation of inorganic SEI products such as LiF, Li_3_N, Li_2_O, and Li_x_S. Ultimately, the observed spectral evolution along with the postmortem XPS data indicates that LiTFSI decomposition primarily contributes to inorganic SEI species formation, improving interfacial stability.

Lithium fluoride (LiF), lithium nitride (Li_3_N), and lithium oxide (Li_2_O) play critical roles in stabilizing the solid electrolyte interphase (SEI) in lithium‐metal and lithium‐ion batteries by enhancing its mechanical strength, ionic conductivity, and uniformity. LiF, with its high surface energy and shear modulus, promotes lithium deposition parallel to SEI, effectively preventing dendrite growth. Its large band gap (8.9 eV) acts as a protective barrier, minimizing electrolyte decomposition and controlling SEI thickness, while its grain boundaries facilitate efficient lithium‐ion diffusion [51, 52, 53]. LiF in the SEI improves the homogeneity of local current distribution by blocking electron leakage and mechanically leveling the interface, thus preventing localized current hotspots that drive dendrite formation. Li_3_N has been shown to enhance SEI stability by introducing lithophilic nitrogen sites that promote uniform lithium‐ion deposition and reduce nucleation overpotential. Its relatively high ionic conductivity (∼10^−^^4^ S cm^−^^1^) facilitates efficient lithium‐ion transport within the SEI, supporting homogeneous ion diffusion and effectively suppressing dendrite growth [54, 55]. Li_2_O, formed from lithium reactions with salt, contributes to a chemically stable and compact SEI, effectively suppressing further electrolyte decomposition and supporting long‐term interfacial stability [52]. Prior studies, primarily based on postmortem or indirect analyses, have proposed that the coexistence of LiF, Li_3_N, and Li_2_O imparts a mechanically flexible and electrochemically resilient SEI. In contrast, our operando FTIR investigation directly captures the dynamic evolution of these inorganic components during cycling, providing time‐resolved insight into their formation and interplay at the Li–electrolyte interface [56].

### 3.3 *In – Operando* FTIR Cell Cycling with DMEDOL – ClO_4_ Electrolyte

**FIGURE 4:**
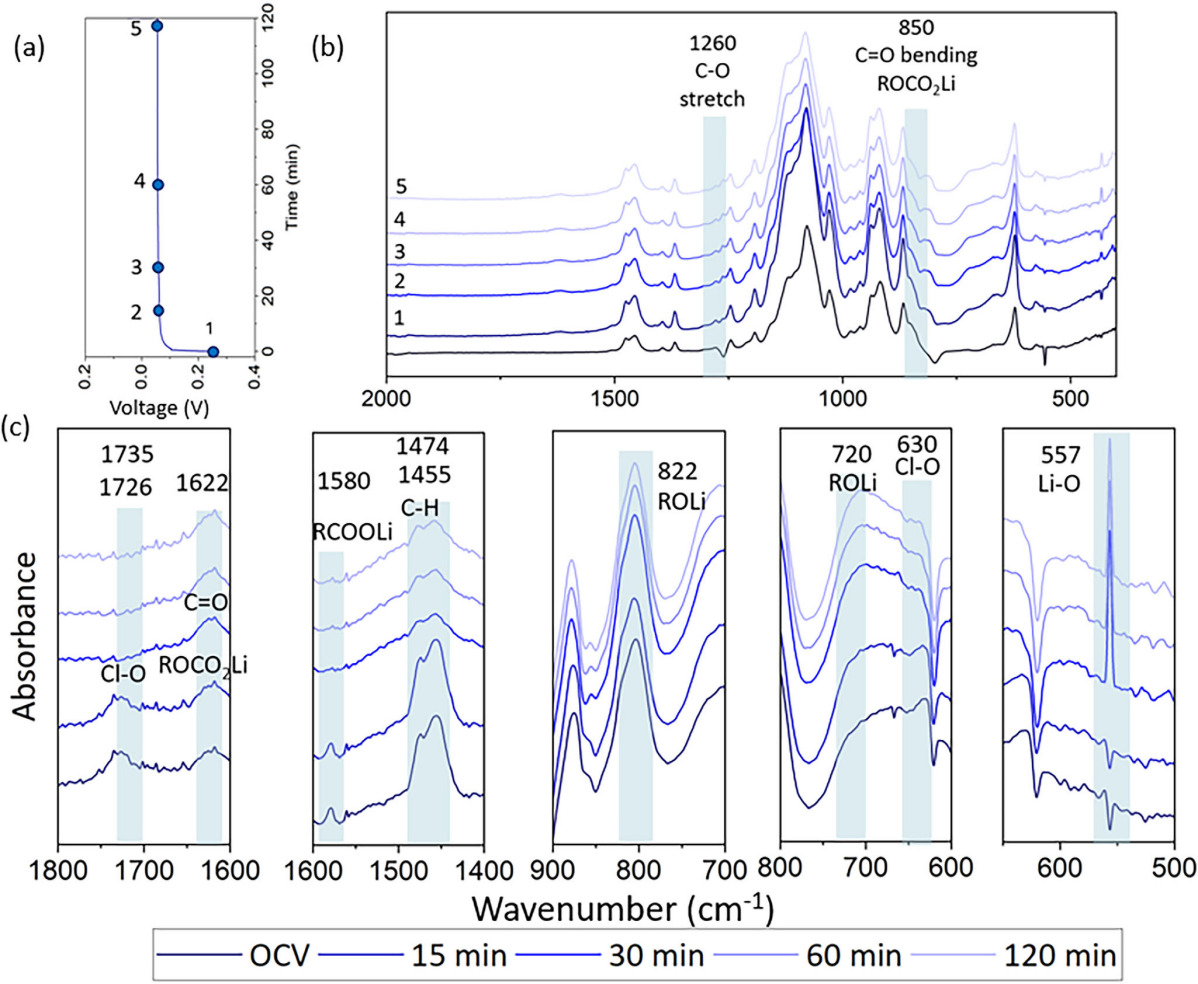
*In‐operando* FTIR spectra during first plating with DMEDOL – ClO_4_ electrolyte (a) Voltage vs time (min) plot during plating (b) FTIR spectra of DMEDOL – ClO_4_, OCV, 15, 30, 60, and 120 min into the plating (c) Magnified views of key vibrational bands, highlighting the emergence and evolution of SEI‐related species.

Figure 4 presents the *in‐operando* FTIR spectra of the Li‖Li symmetric cell using DMEDOL with 1 m LiClO_4_ (DMEDOL – ClO_4_ electrolyte). As shown in Figure 4b, the spectra collected at various time points during plating (OCV, 15 min, 30 min, 60 min, and 120 min) reveal two prominent changes associated with SEI formation: a C─O stretching peak at 1250 cm^−^^1^ and an ROCO_2_Li‐related peak at 850 cm^−^^1^. These signals likely originate from the ring‐opening polymerization of DOL, indicating the formation of organic SEI species. In contrast, when Li metal is exposed to DMEDOL solvent without any salt (Figure 1b), the main features appear at 800 and 1622 cm^−^^1^, corresponding to C═O bending and stretching vibrations, respectively. This difference suggests a distinct DOL reduction pathway in the presence of LiClO_4_, possibly due to the influence of excess Li^+^ ions from the salt.

**FIGURE 5:**
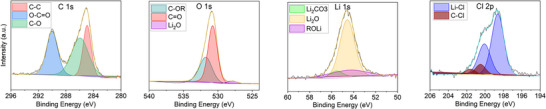
High‐resolution XPS spectra of various elements of lithium cycled in DMEDOL – ClO_4_ electrolyte.

To better isolate the contributions from salt anion in the SEI components, the spectrum of the base DMEDOL – ClO_4_ electrolyte was subtracted, and the resulting difference spectra are shown in Figure 4b,c. Notable salt‐related changes begin to appear after approximately 15 min into the plating process, indicating the progressive breakdown of ClO_4_^−^ anion into species such as Li_2_O, ROLi, and Cl─O. *Postmortem* XPS analysis in Figure 5 further confirms the formation of LiCl, evidenced by the Cl 2p peak at 198.2 eV. A LiCl‐rich SEI has been reported to suppress side reactions between metallic lithium and the electrolyte, while promoting more uniform Li^+^ distribution across the surface, which facilitates homogeneous lithium nucleation and growth. This behavior likely contributes to the improved electrochemical performance discussed later in Figure 8.

LiClO_4_ decomposes at the lithium metal surface under reducing conditions, forming Li_2_O and LiCl as stable SEI components. The ClO_4_^−^ anion undergoes stepwise reduction, releasing oxygen that reacts with Li^+^ to form Li_2_O, while chloride ions combine with Li^+^ to generate LiCl.

LiClO4→Li2O+LiCl

### 3.4 Operando FTIR Cell Cycling with DMEDOL – DFOB Electrolyte

**FIGURE 6:**
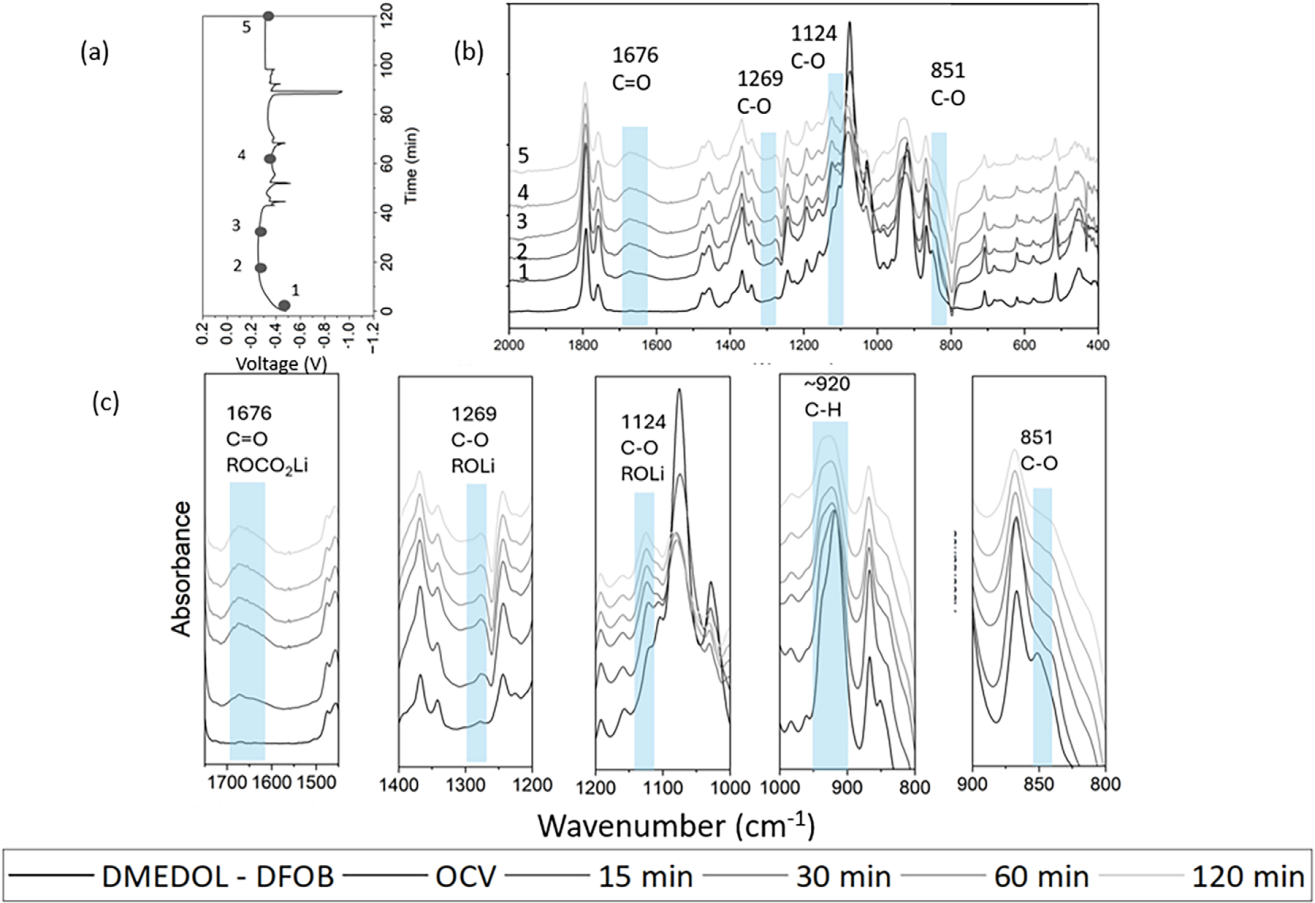
*In‐operando* FTIR spectra during first plating with DMEDOL –DFOB electrolyte (a) Voltage vs time (min) plot during plating (b) FTIR spectra of DMEDOL – DFOB, OCV, 15, 30, 60, and 120 min into the plating (c) Magnified views of key vibrational bands, highlighting the emergence and evolution of SEI‐related species.

The operando FTIR spectra in Figure 6 provides clear evidence that the SEI in the DMEDOL‐DFOB electrolyte system is predominantly organically rich. The prominent C═O stretching peak around 1676 cm^−^^1^ indicates the formation of lithium‐containing carbonyl compounds, probably ROCO_2_Li_,_ likely from the decomposition of DOL [57]. The C─O stretching peaks around 1269 and 1124 cm^−^^1^ suggest the presence of lithiated organic species, including lithiated oligomers (ROLi) [33]. Additionally, the C─H stretching peak near 845 cm^−^^1^ points to the ring‐opening reactions of DOL and further functionalization of the resulting species in the SEI. The wide peak around 920 cm^−^^1^ indicates a poly DOL SEI on lithium surface. Over time, the continuous increase in the intensity of peaks indicating that the SEI continues to develop as plating progresses [42]. Additionally, C 1s spectra shows almost 53.13 at% of carbon compounds are C─O, indicating a significant presence of organic compounds in the interface.

Operando FTIR data indicate that the DMEDOL‐DFOB electrolyte leads to the formation of a predominantly organic SEI, in contrast to the more inorganic‐rich SEIs observed with the other two salts. While an organic‐rich SEI offers flexibility and can better accommodate volume changes during lithium plating and stripping, it often suffers from mechanical instability. The significant expansion and contraction of lithium metal can cause repeated SEI fracture and reformation, resulting in unstable electrochemical behavior and increased overpotential shown in Figure 6a.

After the first plating cycle, no detectable lithium fluoride (LiF) is formed at the interface; instead, the surface is primarily covered by unreacted salt species. This is in stark contrast to the LiTFSI and LiClO_4_ systems, which form LiF and LiCl, respectively, under the same conditions. Figure S4 shows XPS spectra of Li surface after the first plating. Where the Li cycled in DMEDOL – TFSI shows a significant presence of LiF, DMEDOL – LiClO4 shows the presence of LiCl, DMEDOL – DFOB cycled one doesn't particularly show any inorganic SEI compounds on surface.

**FIGURE 7:**
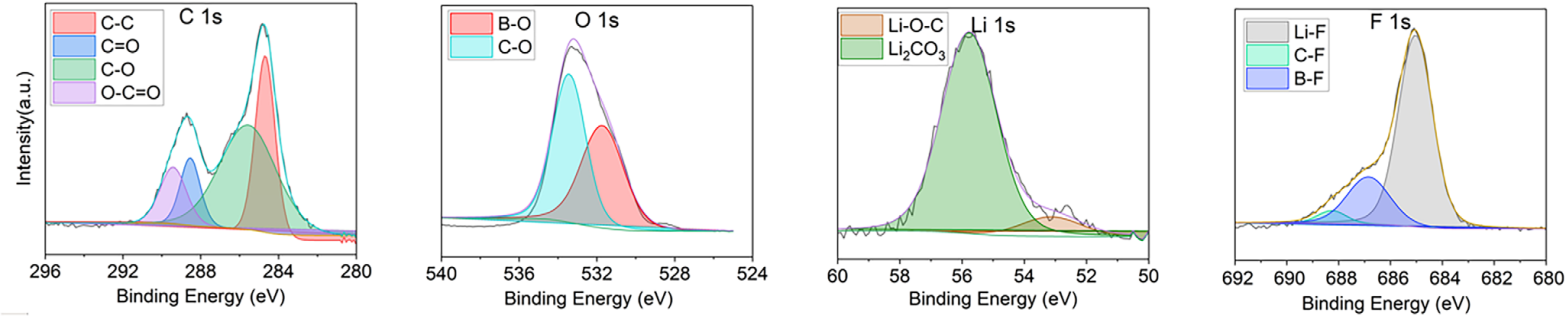
High‐resolution XPS spectra of various elements of lithium cycled in DMEDOL – DFOB electrolyte.

The *postmortem* XPS spectra in Figure 7 shows that after five plating and stripping cycles in DMEDOL – DFOB, inorganic compounds such as LiF and Li_2_CO_3_ begin to gradually appear on the surface. However, the delayed formation and relatively minor presence of these inorganic species suggest they are insufficient to establish a stable SEI, which remains dominated by organic components. This indicates that in ether‐based electrolytes, LiDFOB has limited reactivity with the lithium metal surface, resulting in minimal formation of inorganic species such as LiF and a limited impact on SEI composition.

While LiF has long been recognized for its role in stabilizing SEI layers, the work by He et al., investigating the intrinsic behavior of LiF using model LiF interfaces demonstrated that both ex situ and in situ LiF layers undergo breakdown during cycling. Their findings attributed performance improvements not to LiF itself, but rather to the electrolyte's ability to dynamically regenerate the SEI through continuous decomposition and reformation during operation [58]. In our system, LiDFOB appears to act more as a polymerization initiator, promoting the formation of poly‐DOL‐rich organic SEI layers. These highly organic SEI structures potentially lack mechanical robustness and may fracture easily, exposing fresh lithium surfaces to the electrolyte. This could lead to continuous SEI reformation, increased interfacial instability, and ultimately poor cycling performance. However, if LiDFOB salt is used as an additive or secondary salt, its limited contribution to LiF formation—in conjunction with decomposition products from the primary salt and solvent—may still play a beneficial role in modifying and stabilizing the SEI under appropriately engineered electrolyte conditions [24, 25, 26, 59].

### 3.5 Electrochemical Impedance Spectroscopy (EIS)

**FIGURE 8:**
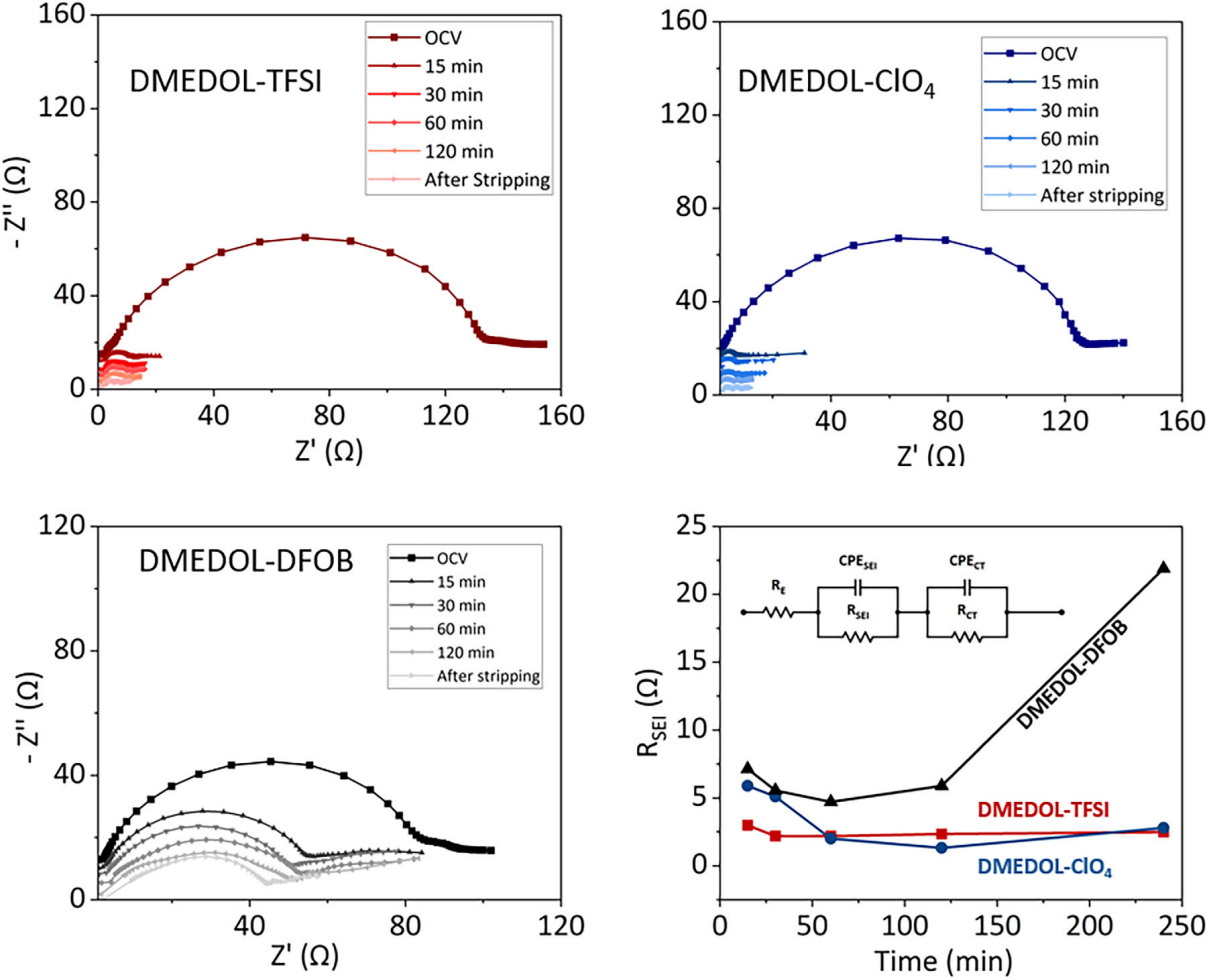
Nyquist plot for Li‖Li symmetric cells with (a) DMEDOL–LiTFSI, (b) DMEDOL–ClO_4_, and (c) DMEDOL–DFOB electrolyte; (d) Time evolution of the fitted SEI resistance (R_s__e__i_) extracted using the equivalent circuit shown in the inset.

Figure 8 shows the Nyquist plot for the three salt electrolytes. The impedance data were analyzed via equivalent circuit fitting to deconvolute contributions from electrolyte resistance, solid electrolyte interface (SEI) transport, and interfacial charge transfer (CT). All spectra were modeled using a consistent equivalent circuit: R_E_ + (R_SEI_ || CPE _SEI_) + (R_CT_ ||CPE_CT_). The use of two interfacial time constants was necessitated by the observation of two spectrally separable arcs in the Nyquist plots, where the high‐frequency response corresponds to transport through the SEI and the lower‐frequency response represents interfacial charge‐transfer kinetics. Constant phase elements (CPEs) were utilized in place of ideal capacitors to account for non‐ideal capacitive behavior arising from surface roughness, porosity, and the inherent heterogeneity of the lithium electrode. Applying the same circuit model across all electrolyte systems and time points enabled a direct, quantitative comparison of extracted parameters while ensuring a robust fit without over‐parameterization.

The extracted R_SEI_ highlights a distinct difference in interfacial stability among the three salt electrolytes. For DMEDOL‐TFSI, resistance increases from 1.26 Ω at OCV to 3 Ω within 15 min and remains nearly constant through 240 min (after stripping). In contrast, the resistance for DMEDOL – ClO_4_ evolves more slowly, stabilizing around 60 min, which correlates with operando FTIR data showing major inorganic SEI components forming after that 30‐min mark. DMEDOL‐DFOB displays the least stable interface, with resistance increasing to 22 Ω after stripping, suggesting significant SEI disruption. Ultimately, the rapid stabilization and minimal post‐stripping perturbation of LiTFSI indicate the formation of a more uniform, ionically conductive, and mechanically robust SEI [60].

**FIGURE 9:**
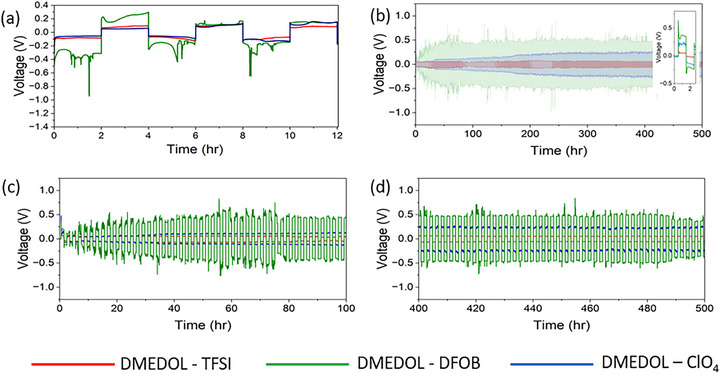
(a) Overpotential vs. time data at 3mA/cm^2^ current density of the FTIR‐ATR cell, (b) 3 mAh/cm^2^ coin cell cycling data (inset zoomed first plating and stripping cycle) (c) 3mA/cm^2^ coin cell cycling data for first 50 h and (d) 3mA/cm^2^ coin cell cycling data for 400–450 h for LiTFSI, LiClO4 and LiDFOB in DMEDOL electrolyte.

Figure 9 presents a comparative evaluation of the electrochemical performance of DMEDOL – TFSI, DMEDOL – ClO_4_, and DMEDOL – DFOB electrolytes during lithium plating and stripping at a current density of 3 mA/cm^2^. Panel (a) shows operando FTIR‐ATR cycling voltage data, while panels (b,c) and (d) display cycling profiles from coin cell tests under identical current density conditions. In all cases, DMEDOL ‐TFSI electrolyte demonstrates the most stable and efficient performance, outperforming both DMEDOL – ClO_4_ and DMEDOL – DFOB electrolyte‐based systems.

During the initial plating process, high overpotentials with sharp voltage spikes were observed, corresponding to the nucleation overpotential—the additional potential required to initiate lithium nucleation, which is typically higher than that needed for subsequent growth. The initial nucleation overpotentials for DMEDOL‐TFSI, DMEDOL – ClO_4_, and DMEDOL – DFOB electrolytes were approximately −0.20 , −0.30, and −0.6 V, respectively. Over time, the overpotentials decreased and stabilized at 0.06 V for LiTFSI and 0.10 V for LiClO_4_. In contrast, the LiDFOB‐based electrolyte exhibited persistently high overpotentials, stabilizing around 0.45 V in coin cells and reaching nearly 1 V in the operando FTIR cell. These results suggest that LiDFOB forms an unstable SEI on the lithium surface, which impedes uniform lithium deposition and contributes to poor electrochemical performance. The galvanostatic cycling data shows high overpotential and unstable performance for DMEDOL – DFOB electrolyte, likely due to the formation of a predominantly organic SEI layer, which lacks the mechanical robustness needed to sustain cycling. This observation directly correlates with the in‐operando FTIR results, which reveal the dominance of organic SEI species, known to be mechanically fragile and susceptible to repeated fracture and reformation—resulting in continuous electrolyte degradation, lithium loss, and increased dendrite formation.

**FIGURE 10:**
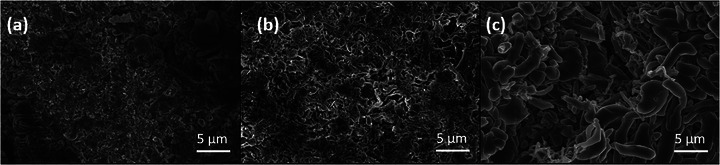
SEM images of postmortem Li metal after plating with (a) DMEDOL – TFSI, (b) DMEDOL – ClO_4,_ and (c) DMEDOL – DFOB electrolyte.

The physical consequences of SEI instability are visualized in the surface morphologies in Figure 10 and Figure S5. After cycling, the DMEDOL‐TFSI surface remains uniform, while the LiDFOB cell exhibits dendritic and mossy deposits. These results highlight how interfacial instability and Li dendrite formation, noted as primary causes for capacity loss and safety issues in lithium‐metal batteries, are heavily influenced by salt chemistry [61]. Cross‐sectional SEM and EDS maps on Figure S5 confirm that the DMEDOL–DFOB system forms a thick, carbon‐rich, and irregular interface. This organic‐heavy SEI is likely mechanically fragile, leading to poor interfacial stability and degraded electrochemical performance observed for LiDFOB‐based electrolytes.

The composition of the SEI is highly dependent on electrolyte salt chemistry and is critical in determining interfacial stability. In the DMEDOL–TFSI system, the SEI comprises inorganic species such as LiF, Li_2_O, and Li_3_N alongside organic components like ROLi and ROCOOLi, resulting in a mechanically robust and ionically conductive interface. Conversely, the DMEDOL– ClO_4_ electrolyte produces delayed passivation and SEI stabilization and results in higher overpotential during extended cycling. For the DMEDOL–DFOB electrolyte, SEI formation is dominated by organic and polymeric species with minimal inorganic contribution, yielding a carbon‐rich, mechanically unstable interface. Research by Khan et al., demonstrated that an interface dominated by carbon‐rich species such as carbonate‐rich ROCOOLi, Li_2_CO_3,_ was insufficient to stabilize the SEI at high rate conditions [62]. This observation is consistent with our findings from operando FTIR and postmortem XPS with DMEDOL – DFOB electrolyte.

To understand the effect of SEI stability beyond symmetric Li‖Li cells and to assess its impact under practical operating conditions, full‐cell electrochemical performance was evaluated using LCO‖Li configurations (Figure S6). The DMEDOL‐TFSI electrolyte delivers an initial discharge capacity of 137 mAh g^−^^1^ and retains 77 mAh g^−^^1^ after 20 cycles, with coulombic efficiency remaining close to 100%, indicating a stable Li metal interface and effective SEI formation. In contrast, the DMEDOL–ClO_4_ electrolyte exhibits rapid capacity fading, with discharge capacity decreasing from approximately 133 to 26 mAh g^−^^1^. This initial capacity is comparable with reported literature [63, 64]. The LCO‖Li cell using the DMEDOL‐DFOB electrolyte failed to cycle stably and is therefore not shown, underscoring the inability of the organic‐rich, carbon‐dominated SEI formed in this system to support stable full‐cell operation.

While previous studies have screened various lithium salts using post‐mortem analysis and cell cycling, their conclusions were based on performance metrics alone and lacked direct evidence of the underlying interfacial chemistry [8, 11]. By employing operando FTIR spectroscopy, to the best of our knowledge, we provide the first direct, real‐time comparison of how the SEI forms and evolves with three different lithium salts. This investigation is conducted at a current density of 3 mA/cm^2^, which more closely resembles practical operating conditions. This approach allows us to forge a direct link between the molecular‐level SEI chemistry and the overall cell performance, offering critical mechanistic insights into what possibly governs interfacial stability in lithium metal batteries.

## 4 Conclusions

In summary, we have investigated the effect of three lithium salts on SEI chemistry using *operando* FTIR. Despite significant progress in electrolyte engineering, real‐time mechanistic insight into the SEI is scarce. To the best of our knowledge, this is the first spectroscopic report that directly captures the complex SEI formation phenomenon between these three salts and the lithium metal surface. Our *in‐operando* FTIR results reveal distinct SEI formation behavior for each salt. The LiTFSI salt in DMEDOL‐TFSI electrolyte promotes the formation of most SEI compounds instantaneously upon contact with lithium metal, with peak intensities increasing gradually thereafter. This robust, synergistic SEI is composed of inorganic species (from salt degradation) and organic moieties (from solvent breakdown). This mechanical resilience and chemical stability due to the hybrid SEI formation contribute directly to improved interfacial stability and the lowest overpotential, significantly enhancing overall battery performance and cycle life. In contrast, the LiClO_4_ electrolyte shows slower kinetics, requiring the combined influence of both potential and time (delaying salt degradation until 30 min into plating) for subsequent salt degradation during SEI formation. The LiDFOB salt proved the least effective in this ether system, showing minimal interaction with lithium metal, forming a highly organic polymeric SEI predominantly from solvent decomposition. This weak interfacial passivation leads to unstable SEI growth and poor cycling performance. This mechanistic insight contributes to a deeper, real‐time understanding of salt‐dependent SEI chemistry and offers a valuable reference point for guiding rational electrolyte design and salt selection strategies for high‐performance lithium metal battery development.

## Conflicts of Interest

The authors declare no conflict of interest.

## Supporting information




**Supporting File**: advs74422‐sup‐0001‐SuppMat.docx.

## Data Availability

The data that support the findings of this study are available from the corresponding author upon reasonable request.
