# Supplementary material for: In‐Operando FTIR Study to Investigate the Effect of Varying Lithium Salts on Solid Electrolyte Interface (SEI) Evolution in Lithium Metal Batteries
Source: Adv Sci (Weinh). 2026 Mar 16;13(24):e23503. doi: 10.1002/advs.202523503 (PMC13116073; doi:10.1002/advs.202523503)
Supplement: Supplementary file 1 — Supporting File: advs74422‐sup‐0001‐SuppMat.docx. [file ADVS-13-e23503-s001.docx]

**Supporting Information**


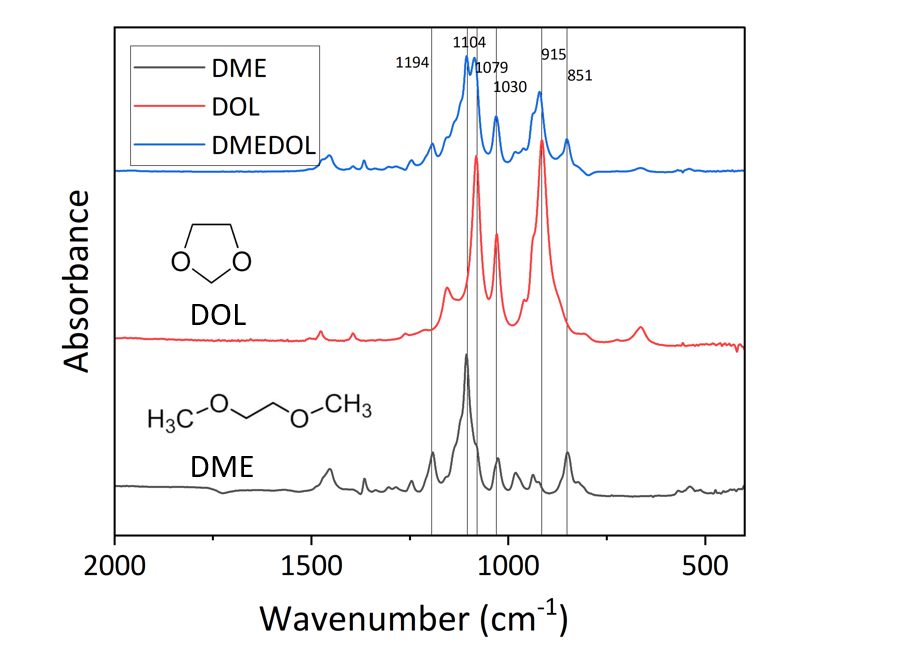


Figure S1 : FTIR spectra of 1,2 – dimethoxyethane (DME), and 1,3 – dioxolane (DOL) solvents

The FTIR spectra of DME (1,2-dimethoxyethane), DOL (1,3-dioxolane), and their 1:1 volumetric solvent mixture (DMEDOL) highlight distinct characteristic vibrational features associated with their molecular structures. The region around 980-1250 cm⁻¹ is dominated by the C-O-C asymmetric stretching vibrations, which are characteristic of ether groups. For DME, this range represents the vibrational modes of its linear ether structure, while for DOL, the peaks in this region arise from the cyclic ether’s C-O-C bonds. The DMEDOL mixture combines both linear and cyclic characteristics, showing overlapping contributions from DME and DOL. A significant peak at 915 cm⁻¹ in the DOL spectrum corresponds to the ring-breathing mode of the cyclic structure, which is absent in the DME spectrum due to its lack of a cyclic framework. This peak persists in the DMEDOL spectrum, confirming the contribution of DOL in the mixture.


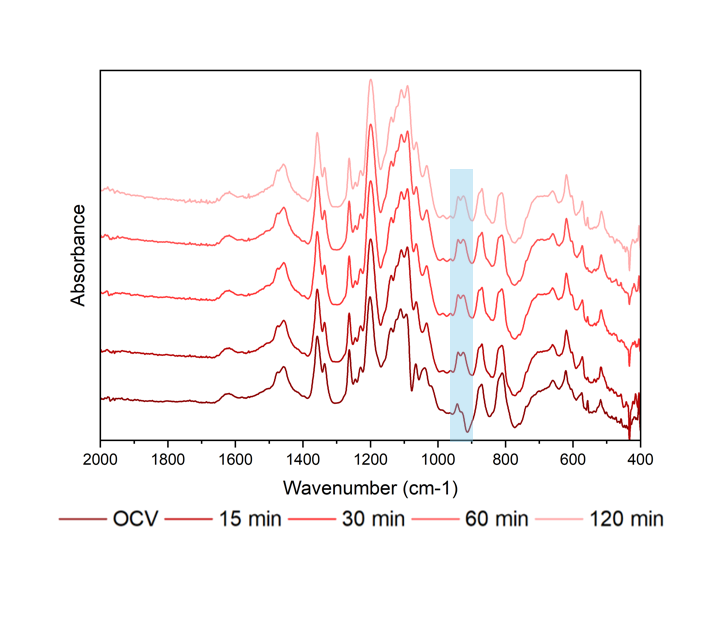


Figure S2: Operando FTIR spectra during first plating with DMEDOL - TFSI electrolyte (Electrolyte subtracted from each spectrum)

Table S1: Peak assignment for the electrolyte subtracted DMEDOL - TFSI operando data

| Peak Position | Peak Assignment |
| --- | --- |
| 571 | Li-O stretching |
| 676 | S-N stretching |
| 811 | ${Li}_{2}{SO}_{3}$ |
| 869 | C=O stretching |
| 920 | C-H bending |
| 1032 | C-O stretching |
| 1107 | C-O-C stretching |
| 1263 | C-O stretching |
| 1335, 1357 | O=S=O stretching |
| 1458 | C=O, Li carbonate |
| 1622 | C=O bending |


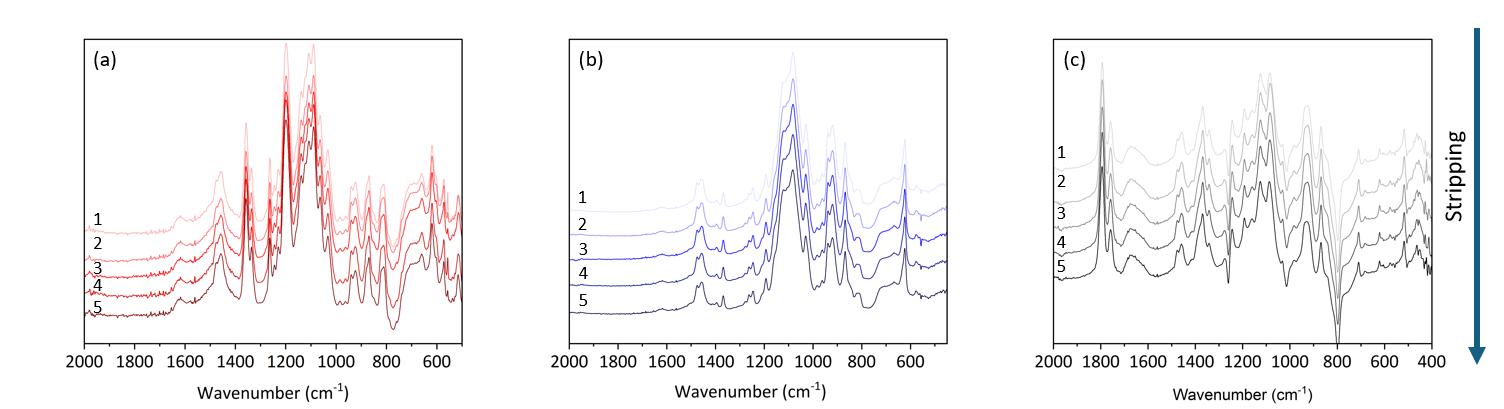


1 – 0 min, 2 – 15 min, 3 – 30 min, 4 – 60 min, 5 – 120 min into the stripping

Figure S3: *In-operando* FTIR spectra during first stripping with a) DMEDOL - TFSI b) DMEDOL -ClO_4_ and c) DMEDOL - DFOB electrolyte

During the stripping process, no new features were observed in the FTIR spectra apart from a gradual decrease in intensity, indicating that SEI formation in this Li||Li symmetric cell is largely irreversible under the studied conditions.


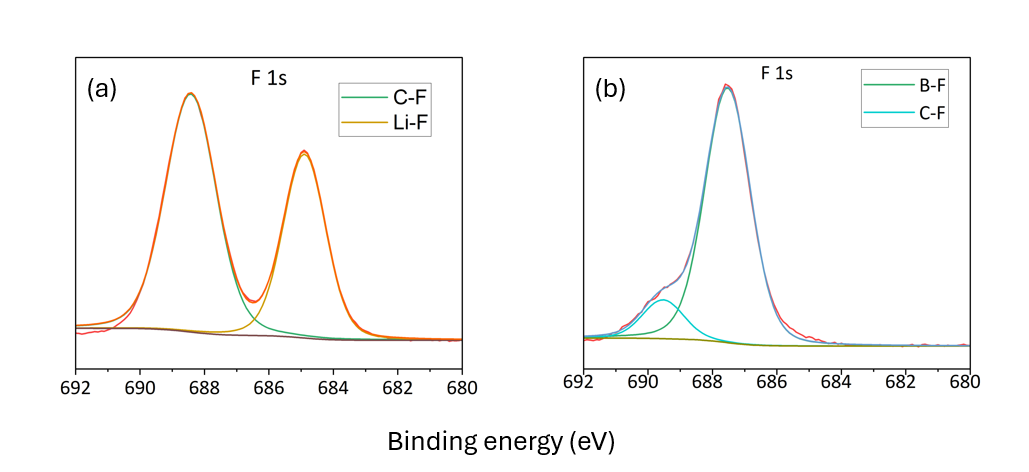


Figure S4: High Resolution XPS spectra of F 1s for (a) DMEDOL – TFSI and (b) DMEDOL – DFOB electrolyte after first plating

Table S2: Extracted R_E_, R_SEI_, R_CT_ from equivalent circuit plot

| **Electrolyte** | **Time** | **R_E_** | **R_SEI_** | **R_CT_** |
| --- | --- | --- | --- | --- |
| DMEDOL - TFSI | OCV | 3.406 | 1.262 | 138 |
|  | 15min | 2.596 | 2.995 | 7.959 |
|  | 30 min | 2.377 | 2.197 | 7.637 |
|  | 60 min | 2.377 | 2.197 | 7.637 |
|  | 120 min | 2.491 | 2.848 | 6.305 |
|  | 240 min | 3.098 | 2.493 | 5.994 |
| DMEDOL – ClO_4_ | OCV | 2.5 | 1 | 124 |
|  | 15min | 2.025 | 5.9 | 0.74374 |
|  | 30 min | 2.039 | 5.106 | 0.678 |
|  | 60 min | 2.115 | 2.017 | 2.59 |
|  | 120 min | 2.37 | 1.319 | 2.72 |
|  | 240 min | 3.52 | 2.8 | 3.081 |
| DMEDOL – DFOB | OCV | 2.779 | 9.14 | 82.55 |
|  | 15min | 3.127 | 7.139 | 52.55 |
|  | 30 min | 3.765 | 5.564 | 47.51 |
|  | 60 min | 4.35 | 4.716 | 50.43 |
|  | 120 min | 4.683 | 5.897 | 46.71 |
|  | 240 min | 4.972 | 21.9 | 19.82 |


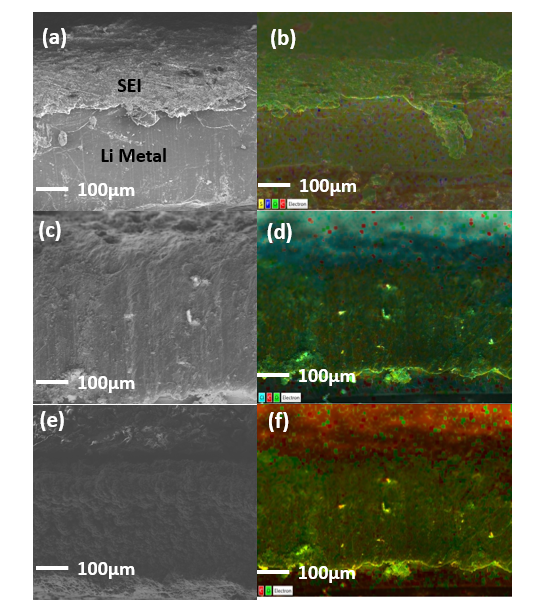


Figure S5: Cross-sectional SEM and corresponding SEM–EDS elemental maps of post-mortem lithium metal electrodes after cycling in (a,b) DMEDOL–TFSI, (c,d) DMEDOL–ClO₄, and (e,f) DMEDOL–DFOB electrolytes

**
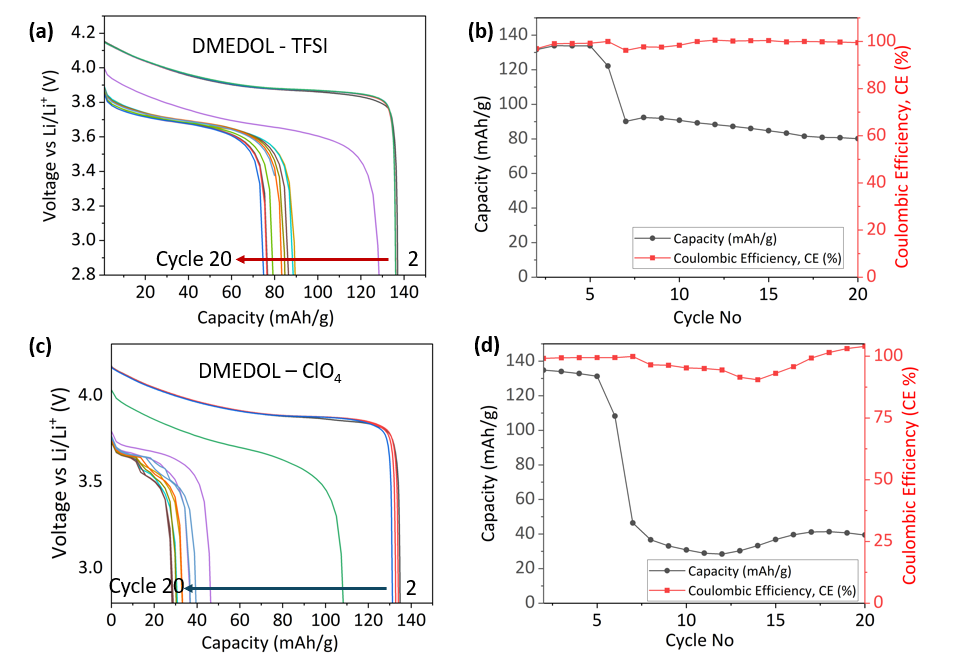
**

Figure S6: Electrochemical performance of LCO‖Li cells cycled in (a,b) DMEDOL–TFSI and (c,d) DMEDOL–ClO₄ electrolytes. Panels (a) and (c) show voltage–capacity profiles at selected cycles, while panels (b) and (d) present the corresponding discharge capacity and Coulombic efficiency as a function of cycle number. Cells were initially cycled at 0.1 C for 5 formation cycles, followed by cycling at 1 C
